# ﻿Four new *Phragmidium* (Phragmidiaceae, Pucciniomycetes) species from Rosaceae plants in Guizhou Province of China

**DOI:** 10.3897/mycokeys.93.90861

**Published:** 2022-11-10

**Authors:** Jing-E Sun, Qian Zhang, Wen-Mei Luo, Yuan-Qiao Yang, Hua-Ming An, Yong Wang

**Affiliations:** 1 Department of Plant Pathology, Agricultural College, Guizhou University, Guiyang, 550025, China; 2 Agricultural College, Guizhou University, Guiyang, 550025, China; 3 Guizhou Engineering Research Center for Fruit Crops, Guiyang, 550025, China

**Keywords:** Basidiomycota, ITS, LSU, phylogeny, rust disease, taxonomy

## Abstract

In this study, four new species of *Phragmidium* were proposed based on morphological and molecular characters. In morphology, *Phragmidiumrosae-roxburghii* sp. nov. was distinguished to related taxa by its unique square to diamond-shaped urediniospores; *Ph.rubi-coreani* sp. nov. differed from *Ph.barclayi* and *Ph.cibanum* because of teliospores with fewer cells and shorter pedicels; urediniospores of *Ph.potentillae-freynianae* sp. nov. were bigger than *Ph.duchesneae-indicae*; and *Ph.rosae-laevigatae* sp. nov. produced bigger urediniospores than *Ph.jiangxiense*. The phylogenetic analyses based on the combination of two loci (ITS and LSU) also supported our morphological conclusion. In the meantime, three previously known species were also described herein.

## ﻿Introduction

*Phragmidium* (Phragmidiaceae) was established by Link (1816) and characterized by laterally separated multicellular teliospores with pigmented bilaminar walls, and a thickened pedicel at the base (Wei 1988).

The genus was widely distributed around the world especially in the northern hemisphere, such as China, USA and Japan (Wei 1988; Zhuang 1989; Cummins and Hiratsuka 2003; Maier et al. 2003; Zhuang et al. 2012; Pscheidt and Rodriguez 2016; Liu et al. 2018, 2019, 2020; Zhao et al. 2021). *Phragmidium* species often caused severe rust diseases in Rosaceae plants (*Rosa*, *Rubus*, *Potentilla*, *Sanguisorba*, *Duchesnea* and *Acaena*). Species of *Phragmidium* have been reported growing on host plants of *Rosa*, *Rubus*, and *Potentilla*, with a few species on *Sanguisorba* (Cummins and Hiratsuka 2003; Maier et al. 2003; Yun et al. 2011; Pscheidt and Rodriguez 2016; Liu et al. 2018, 2019, 2020), *Duchesnea* (Zhao et al. 2021) and *Acaena* (McTaggart et al. 2016). Two species *Ph.mucronatum* (Pers.) Schltdl. and *Ph.tuberculatum* Jul. Müll., were common pathogens on ornamental roses worldwide (Wahyuno et al. 2001, 2002; Leen and Van Huylenbroeck 2007; Wilson and Aime 2014).

About 8000 species of rust fungi have been reported in the world (Zhao et al. 2021). Based on morphological features or host associations, 1200 species belonging to 71 genera of 15 families were previously reported in China. Over 70 *Phragmidium* species have been described (Cummins 1931; Arthur 1934; Zhuang et al. 1998, 2003, 2005, 2012; Wahyuno et al. 2001; Cummins and Hiratsuka 2003; Yang et al. 2015; Ali et al. 2017; Aime et al. 2018; Liu et al. 2018, 2019, 2020; Ono and Wahyuno 2019; Aime and McTaggart 2021; Zhao et al. 2021).

Traditionally, *Phragmidium* species are distinguished based on teliospores morphology (Wei 1988). According to Wahyuno et al. (2001) and Zhao et al. (2021)*Phragmidium* species are difficult to distinguish based only on morphology of asexual spore stages; thus, DNA data is essential for taxonomy and identification of *Phragmidium* species.

The combination of morphological and molecular characters has been applied in the taxonomy of rust fungi (Beenken et al. 2012; Beenken 2014; McTaggart et al. 2016, 2017; Liu et al. 2018, 2019, 2020; Ono and Wahyuno 2019; Zhao et al. 2021). *Phragmidium* includes more than 270 epithet records which are listed in MycoBank (https://www.mycobank.org) and Index Fungorum (http://www.indexfungorum.org) (accessed in October 2022). However, only 28 records were described and named by Chinese researchers, three *Phragmidium* taxa in Guizhou Province, *Ph.duchesneae-indicae*, *Ph.nonapiculatum* and *Ph.kans* were introduced by Zhao et al. (2021). In the present study, thirteen fresh rust specimens were collected on eight Rosaceae hosts, such as *Duchesneaindica*, *Potentillafreyniana*, *P.kleiniana*, *Rosaroxbunghii*, *R.laevigata*, *Rosa* sp., *Rubuscoreanus* and *Ru.parrifolius* in Guizhou Province. This study aimed to determine the taxonomic status of the parasitic pecies of the Rosaceae in Guizhou Province through morphological and molecular characters. Meanwhile, we hope to contribute a significant amount of molecular data that may aid future studies and phylogenetic placement of *Phragmidium* in the Pucciniales.

## ﻿Materials and methods

### ﻿Sampling and microscopy observation

Thirteen fresh rust specimens were collected on branch and leaf from eight species of Rosaceae, *Duchesneaindica*, *Potentillafreyniana*, *P.kleiniana*, *Rosaroxbunghii*, *R.laevigata*, *Rosa* sp., *Rubuscoreanus* and *R.parrifolius* in Guizhou Province, China. The spores from specimens were mounted in sterile water, on slides and observed using a Zeiss Scope 5 compound microscope (Axioscope 5, Jena, Germany), and photographed with an AxioCam 208 color (Jena, Germany) camera and saved as JPG files. Approximately 30 measurements were made of each feature using the ZEN 2.0 (blue edition) software. The Flora of China (http://www.efloras.org/flora_page.aspx?flora_id=4) was used to identify host plants (Liu et al. 2018). The rust specimens were deposited in the HGUP Herbarium of Department of Plant Pathology, Agricultural College, Guizhou University. Taxonomic details of our novel taxa were submitted to MycoBank (www.mycobank.org).

### ﻿DNA extraction, PCR and sequencing

Rust spores were scraped from fresh plant tissues using a sterile scalpel. Total DNA of rust spores was extracted with a BIOMIGA Fungus Genomic DNA Extraction Kit (GD2416) according to the manufacturer’s protocol. Targeted sequences of internal transcribed spacer of rDNA (ITS) was amplified using primers ITS4rust (5’-CAGATTACAAATTTGGGCT-3’) (Beenken et al. 2012) and Rust2inv (5’-GATGAAGAACACAGTGAAA-3’) (Aime 2006), and the large subunit (LSU) of the ribosomal RNA gene was amplified using the primers No.4 (5’-ACCCGCTG AATTTAAGCATAT-3’)/No.11 (5’-CTCCTTGGTCCGTGTTTCAAGACGC-3’) (Van der Auwera et al. 1994), or LR6 (5’-CGCCAGTTCTGCTTACC-3’) (Vilgalys and Hester 1990), and LR0R (5’-ACCCGCTGAACTTAAGC-3’) (Hopple 1994). The PCR cycling conditions were as described by Liu et al (2018). The PCR amplicons from purification and sequencing were carried out at Sangon Biotech (Chengdu, China). Newly-generated sequences were deposited in GenBank (Table 1).

**Table 1.** Specimens and GenBank accession numbers of rust isolates included in this study.

Species	Voucher specimens	Host	Locality	ITS	*LSU*
*Phragmidiumandersoni*	HMAS-53231 ^T^	*Potentillafruticosa*	Sinkiang, China	N/A	MG669120
*Ph.altaicum*	BJFCR03247	*Rosaalbertii*	China	MH285385	MH285381
	BJFCR03246	*Rosaalbertii*	China	MH285384	MH285380
	BJFCR03217 ^T^	*Rosaalbertii*	China	MH285383	MH285379
*Ph.barclayi*	HMAS-67281	*Rubusaustrotibetanus*	Tibet, China	N/A	MG669117
*Ph.barnardii*	BRIP 56945	*Rubusparvifolius*	South Africa	N/A	KT199402
***Ph.barnardii***	**HGUP21035**	***Rubusparvifolius***	**Guizhou, China**	**OL684828**	**OL684839**
*Ph.biloculare*	BPI:881121	*Potentillaflabellifolia*	USA	N/A	JF907670
*Ph.butleri*	HMAS-67841	*Rosamacrophylla*	Tibet, China	N/A	MG669118
*Ph.chayuensis*	BJFC-R02532 ^T^	*Rosaduplicata*	Tibet, China	N/A	MG669112
	BJFC-R03014 ^T^	*Rosaduplicata*	Tibet, China	N/A	MG669113
*Ph.cibanum*	BJFCR02528 ^T^	*Rubusniveus*	Tibet, China	MH128370	MG669110
	BJFCR03012 ^T^	*Rubusniveus*	Tibet, China	MH128371	MG669111
***Ph.duchesneae-indicae***	**HGUP21031**	***Duchesneaindica***	**Guizhou, China**	**OL684824**	**OL684835**
	**HGUP21032**	***Duchesneaindica***	**Guizhou, China**	**OL684825**	**OL684836**
*Ph.fragariae*	WM 1317	*Potentillasterilis*	Europe	N/A	AF426217
*Ph.fusiforme*	T-10	*Rosapendulina*	Switzerland	N/A	AJ715522
*Ph.fructigenum*	HMUT100472	*Rosaglomerata*	Guangdong, China	N/A	KU059168
*Ph.griseum*	BJFCR03449	*Rubuscrataegifoliu*	Beijing, China	MN264712	MN264730
	BJFCR03451	*Rubuscrataegifoliu*	Beijing, China	MN264713	MN264731
	HMAS56906	*Rubuscrataegifoliu*	Beijing, China	N/A	MG669115
*Ph.handelii*	BJFC-R01030	*Rosawebbiana*	Gansu, China	N/A	KP407631
*Ph.ivesiae*	BPI-877968	*Potentillagracilis*	USA	N/A	JF907673
	BPI-863637	*Potentillagracilis*	USA	N/A	JF907672
	BJFC-R01421	*Rosawebbiana*	Gansu, China	N/A	KP407628
*Ph.japonicum*	HMAS41585	*Rosalaevigata*	Fujian, China	MN264716	MN264734
	IBAR8174	*Rosaluciae*	Ibaraki, Japan	MN882389	MN848143
*Ph.jiangxiense*	BJFCR03452	*Rosalaevigata*	Jiangxi, China	MN264714	MN264732
	BJFCR03453 ^T^	*Rosalaevigata*	Jiangxi, China	MN264715	MN264733
*Ph.leucoaecium*	BJFCR02116	*Rosa* sp.	Yunnan, China	MN264718	MN264736
	BJFCR02118 ^T^	*Rosa* sp.	Yunnan, China	MN264719	MN264737
*Ph.longissima*	BJFC-R00338	*Rosalichiangensis*	Yunnan, China	N/A	KP407633
	BJFC-R00360	*Rosalichiangensis*	Yunnan, China	N/A	KP407634
*Ph.mexicanum*	BPI 843961	*Potentillaindica*	Maryland, USA	N/A	JF907660
	BPI 843829	*Potentillaindica*	Virginia, USA	N/A	JF907659
*Ph.mucronatum*	RUBO	*Rosa* sp.	Bochum, Germany	N/A	KU059171
	TUB 012090	*Rosacorymbifera*	Germany	N/A	AJ715520
*Ph.montivagum*	HMAS67176	*Rosadavurica*	China	N/A	KU059173
	FO 47828	*Rosawoodsii*	NA	N/A	AF426213
*Ph.octoloculare*	HMAS-140416	*Rubusbiflorus*	Tibet, China	N/A	MG669119
*Ph.potentillae*	HMAS53236	*Potentillavirgata*	Sinkiang, China	N/A	MG669114
	BJFCR00961	*Potentillachinensis*	Qinghai, China	MN264720	MN264738
***Ph.potentillae***	**HGUP21034**	***Potentillakleiniana***	**Guizhou, China**	**OL684827**	**OL684838**
*Ph.potentillae-canadensis*	BPI877886	*Potentilla* sp.	North Carolina, USA	N/A	JF907667
	BPI877885	*Potentillacanadensis*	Maryland, USA	N/A	JF907668
***Ph.potentillae-freynianae***	**HGUP21033 ^T^**	***Potentillafreyniana***	**Guizhou, China**	**OL684826**	**OL684837**
*Ph.punjabense*	BA-65A ^T^	*Rosabrunonii*	Pakistan	N/A	KX358854
	BA-65B	*Rosabrunonii*	Pakistan	N/A	KX358855
***Ph.rosae-laevigatae***	**HGUP21036 ^T^**	***Rosalaevigata***	**Guizhou, China**	**OL684829**	**OL684840**
	**HGUP21037**	***Rosalaevigata***	**Guizhou, China**	**OL684830**	**OL684841**
*Ph.rosae-multiflorae*	HMAS71053	*Rosamultiflora*	Shanxi, China	N/A	KU059174
	HMAS94924	*Rosamultiflora*	Zhejiang, China	N/A	KU059175
	BJFCR03454	*Rosamultiflora*	Jiangxi, China	MN264721	MN264739
***Ph.rosae-roxburghii***	**HGUP21025 ^T^**	***Rosaroxburghii***	**Guizhou, China**	**OL684818**	**OL684831**
	**HGUP21026**	***Rosaroxburghii***	**Guizhou, China**	**OL684819**	**OL684832**
	**HGUP21027**	***Rosaroxburghii***	**Guizhou, China**	**OL684820**	**N/A**
	**HGUP21028**	***Rosa* sp.**	**Guizhou, China**	**OL684821**	**OL678103**
*Ph.rosae-rugosae*	BJFCR03455	*Rosarugosa*	Jiangxi, China	MN264722	MN264740
	BJFCR03456	*Rosarugosa*	Beijing, China	MN264723	MN264741
*Ph.rubi-idaei*	WM 1024	*Rubusidaeus*	Europe	N/A	AF426215
	BRIP 59372	*Rubusidaeus*	Australia	N/A	MW147044
*Ph.rubi-oldhami*	HMAS-64306	*Rubuspungens*	Sichuan, China	N/A	MG669116
***Ph.rubi-corean***	**HGUP21029 ^T^**	***Rubuscoreanus***	**Guizhou, China**	**OL684822**	**OL684833**
	**HGUP21030**	***Rubuscoreanus***	**Guizhou, China**	**OL684823**	**OL684834**
*Phragmidium* sp.	HMAS41561	*Rosamultiflora*	Fujian, China	MN264717	MN264735
*Ph.sanguisorbae*	BPI 872232	*Sanguisorbaminor*	USA	N/A	JF907674
	ML 957	*Sanguisorbaminor*	Europe	N/A	AF426216
*Ph.tormentillae*	BPI 843392	*Potentilla* sp.	Maryland, USA	DQ354553	DQ354553
	BPI 877888	*Potentillasimplex*	Tennessee, USA	N/A	JF907669
*Ph.tuberculatum*	BJFCR00959	*Rosa* sp.	Qinghai, China	N/A	KP407636
	BPI 877978	*Rosa* sp.	California, USA	N/A	KJ841919
	BPI 843677	*Rosa* sp.	Argentina	N/A	KJ841921
*Ph.violaceum*	MCA2782	*Rubus* sp.	France	DQ142909	DQ142909
	BPI 871510	*Rubus* sp.	Oregon, USA	DQ142910	DQ142910
	BJFCR03457	*Rubus* sp.	New Zealand	MN264724	MN264742
*Ph.warburgianum*	BJFCR03458	*Rosabracteata*	Japan	MN264726	MN264744
	BJFCR03459	*Rosabracteata*	Japan	MN264727	MN264745
*Ph.zangdongii*	BJFCR02447 ^T^	*Rosatibetica*	Tibet, China	MH128372	MG669108
	BJFCR03013 ^T^	*Rosatibetica*	Tibet, China	MH128373	MG669109
*Ph.zhouquensis*	BJFCR01516 ^T^	*Rosaomeiensis*	Yunnan, China	MN264728	MN264746
	BJFCR01529 ^T^	*Rosaomeiensis*	Yunnan, China	MN264729	MN264747
*Trachysporaintrusa*	BPI 843828	*Alchemillavulgaris*	Switzerland	DQ354550	

^T^ = Type specimens. New specimens are in bold typeface.

### ﻿Phylogenetic analyses

81 sequences, including originated from thirteen specimens and related sequences of *Phragmidium* spp. were aligned in the online version of MAFFT v. 7.307 (Katoh and Standley 2016). *Trachysporaintrusa* (BPI 843828) was selected as outgroup (Liu et al. 2020). The alignment document was edited using MEGA6 (Tamura et al. 2013) and manually adjusted when necessary.

All relevant sequences of ITS—*LSU* dataset were conducted using maximum likelihood (ML), maximum parsimony (MP) and Bayesian inference (BI) methods. ML analysis was performed using RAxML-HPC2 v.8.2.12 (Stamatakis 2014). Gaps were treated as “missing”. The MP analysis of the two loci (ITS and *LSU*) was implemented with PAUP v. 4.0b10 (Swofford 2002). The phylogenetic trees were generated using the heuristic search option with tree bisection reconnection (TBR) branch swapping and 1000 random sequence additions. The maxtrees was set to 5000. The tree length (TL), consistency index (CI), homoplasy index (HI), retention index (RI), and rescaled consistency index (RC) were calculated. Bayesian inference analysis was inferred by MrBayes 3.2.6 (Ronquist et al. 2012). The best model for two loci (ITS and *LSU*) was determined by MrModeltest v2 (Nylander 2004), ITS: HKY+G, *LSU*: GTR+I+G. BI were performed by six Markov chain Monte Carlo. These chains were run for 5 million generations, sampling tree every 100 generations. The first 25% of resulting trees were discarded as burn-in phase of each analysis, and trees were saved every 5000 generations. Alignment matrices have been uploaded as an attachment.

## ﻿Results

### ﻿Phylogenetic analyses

**Figure 1.**
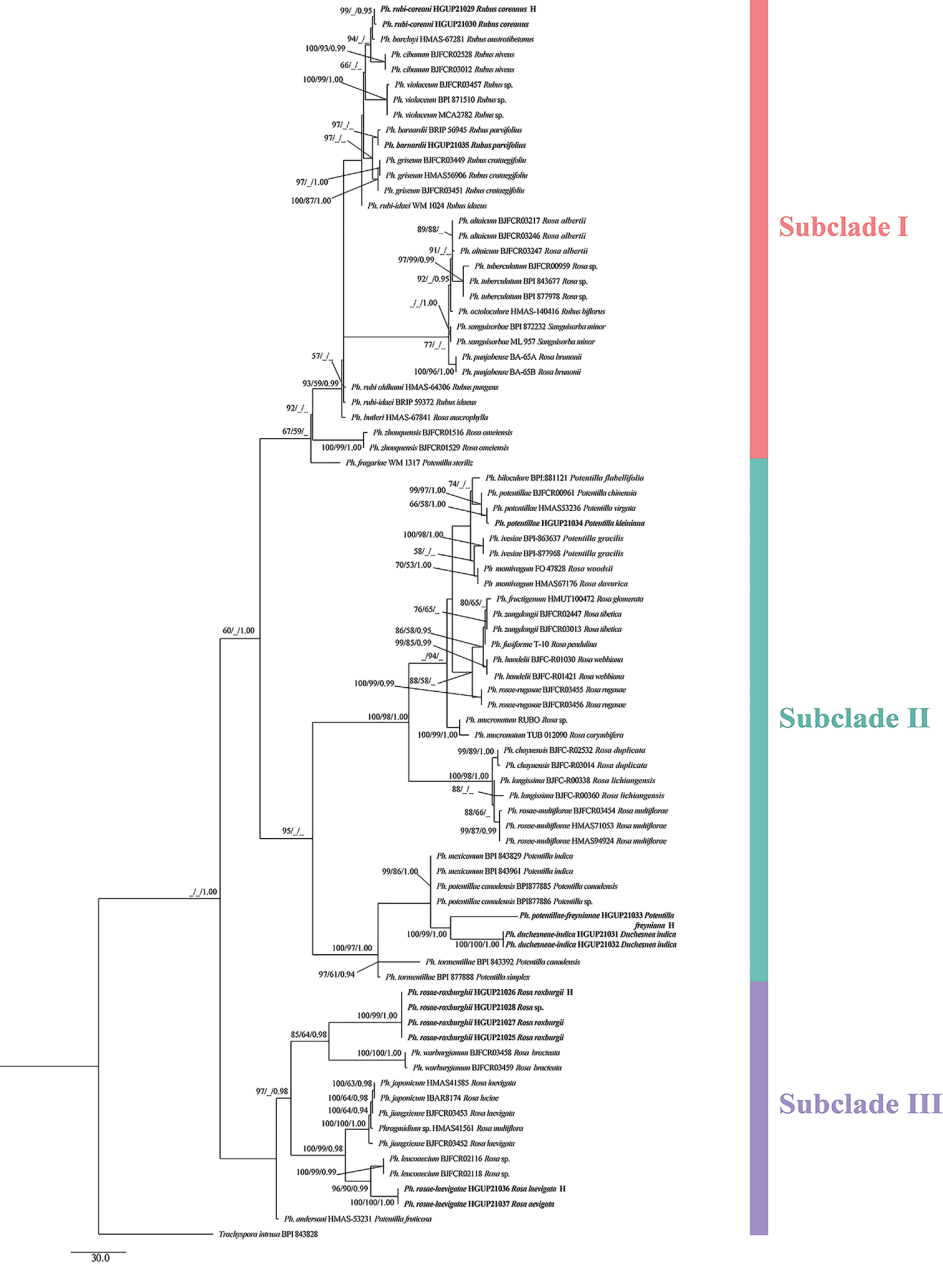
The maximum parsimony tree of 42 *Phragmidium* taxa based on ITS and *LS*U genes; host plants are also given.

The phylogenetic trees accommodated 82 sequences listed in Table 1. The combined alignment including ITS (493 bp) and *LSU* (544 bp) regions consisted of 1067 characters, of which 585 were constant, 89 variable characters were parsimony uninformative, and 363 were parsimony informative. We built three phylogenetic trees, ML tree, MP tree and BI tree. The MP tree was selected to represent the phylogenetic relationship of different *Phragmidium* taxa (Fig. 1). MP analysis produced the following parameters: tree length (TL) = 1011; consistency index (CI) = 0.643; homoplasy index (HI) = 0.356; retention index (RI) = 0.898; and rescaled consistency index (RC) = 0.578. *Phragmidiumrubi-coreani* on *Rubuscoreanus* with telial, aecial and uredinial stages formed a small branch only. *Phragmidiumpotentillae-freynianae* and *Ph.duchesneae-indicae* constituted a distinct subclade with high statistical support (100 ML/99 MP/1.00 PP). *Phragmidiumrosae-laevigatae* was phylogenetically sister to *Ph.leucoaecium*, *Ph.japonicum*, *Ph.jiangxiense* and *Phragmidium* sp. with high support (100 ML/100 MP/1.00 PP). The four aecial-uredinial fungi on *Ro.roxburghii* kept identical base composition on ITS and *LSU* gene regions and made up a distinct subclade to *Ph.warburgianum* with high support (100 ML/99 MP/1.00 PP). Our strains represented four novel taxa, which was also supported by comparison of the DNA base pair differences between our strains and related taxa on ITS and *LSU* gene region.

The hosts of the *Phragmidium* species were mainly concentrated in *Rosa*, *Rubus* and *Potentilla* of Rosaceae (Fig. 1). Eighty-one *Phragmidium* strains clustered together as a clade, which was roughly divided into three subclades (Subclade I, Subclade II and Subclade III). For Subclade I with 16 species (*Ph.rubi-coreani*, *Ph.barclayi*, *Ph.cibanum*, *Ph.violaceum*, *Ph.barnardii*, *Ph.griseum*, *Ph.rubi-idaei*, *Ph.altaicum*, *Ph.tuberculatum*, *Ph.octoloculare*, *Ph.sanguisorbae*, *Ph.punjabense*, *Ph.rubi-oldhami*, *Ph.butleri*, *Ph.zhouquensis* and *Ph.fragariae*) (67 ML/59 MP), their hosts belonged to *Rosa*, *Rubus*, *Potentilla*, and *Sanguisorba*. *Phragmidiumrubi-coreani* and *Ph.rubi-ideai* associated with host plants on the generic level had obvious genetic distance. Subclade II included 18 *Phragmidium* taxa (*Ph.biloculare*, *Ph.potentillae*, *Ph.ivesiae*, *Ph.montivagum*, *Ph.fructigenum*, *Ph.zangdongii*, *Ph.fusiforme*, *Ph.handelii*, *Ph.rosae-rugosae*, *Ph.mucronatum*, *Ph.chayuensis*, *Ph.longissima*, *Ph.rosae-multiflorae*, *Ph.mexicanum*, *Ph.potentillae-canadensis*, *Ph.potentillae-freynianae*, *Ph.duchesneae-indicae* and *Ph.tormentillae*) (95 ML), but their host plants only referred to *Rosa*, *Potentilla* and *Duchesnea*. *Phragmidiumpotentillae-freynianae* and *Ph.duchesneae-indicae* belonging to different generic host plants were accommodated to a branch (100 ML/99 MP/1.00 PP), but *Ph.mexicanum* and *Ph.potentillae-canadensis* formed a clade (99 ML/86 MP/1.00 PP) separated from *Ph.potentillae-freynianae* with the congeneric host plants. *Phragmidiumtormentillae* associated with *Potentillacanadensis* (*P.simplex*) as its host formed an independent branch (97 ML/61 MP/0.94 PP). The *Phragmidium* host plants in Subclade III (*Ph.rosae-roxburghii*, *Ph.warburgianum*, *Ph.japonicum*, *Ph.jiangxiense*, *Phragmidium* sp., *Ph.leucoaecium*, *Ph.rosae-laevigatae*, *Ph.andersoni*) belonged to *Rosa* and *Potentilla*. *Phragmidiumrosae-laevigatae* and *Ph.rosae-roxburghii* with the same generic host plants did not group together (97 ML /0.98 PP). *Phragmidiumjaponicum*, *Ph.jiangxiense* and *Phragmidium* sp. (HMAS51561) all from *Rosa* formed a branch (100 ML/100 MP/1.00 PP). *Phragmidiumandersoni* collected from *Potentillafruticosa* formed an independent branch.

RA×ML and MP bootstrap support values (MP ≥ 50%), and Bayesian posterior probability (PP ≥ 0.90) are marked on the nodes as (ML/MP/PP). Specimens from current study have put in bold and put an H in the selected holotypes. The outgroup was *Trachysporaintrusa* (BPI 843828). The scale bar indicates 30 expected changes per site.

### ﻿Taxonomy

#### ﻿ Phragmidium rosae-roxburghii

Taxon classificationFungiPuccinialesPhragmidiaceae

J.E. Sun & Yong Wang bis sp. nov.

9EA22E28-68E0-517A-80B1-1D74F22C6B79

845041

Figs 2 , 3

##### Diagnosis.

*Phragmidiumrosae-roxburghii* easily to be distinguished by its unique square to diamond-shaped urediniospores.

##### Holotype.

China. Guizhou Province, Panzhou city, 25°89'61"N, 104°56'07"W, 750 m, 21 Mar 2021, on *Rosaroxburghii*, coll. J.E. Sun & Y.Q. Yang, HGUP21025, ITS: OL684818, LSU: OL684831.

##### Etymology.

Referring to the host, *Rosaroxburghii*, on which the fungus was first found.

##### Description.

***Spermogonia***: unknown. ***Aecia*** formed on gold distinct, circular lesions on both sides of the stems, petioles and leaves, rarely produced on the abaxial leaf surface, scattered, flat oval to subglobose, powdery, 1.0–5.0 mm diam. Aeciospores formed in basipetal succession, oval o subglobose, 22–30 × 14–22 µm (mean 26 × 18 µm, n = 30), inclusions golden, to bright-yellow; wall 1.8–3.1 µm thick, colorless, mostly with irregularly elongated verrucae on the surface. ***Uredinia*** produced on the abaxial leaf surface, scattered to gregarious, hypophyllous, orange-colored or white, powdery, oval to rounded, 0.1–1.0 mm diam, paraphysis in the periphery of the uredinia, curved, 30–55 × 9–20 µm, colorless thin-walled. Urediniospores generally angular, square to diamond-shaped, yellowish to orange-colored, 20–30 × 16–21 µm (mean: 25 × 19 µm, n = 30), thick-walled, 0.5–2.0 µm thick, colorless, regularly echinulate with stout spines.

**Figure 2.**
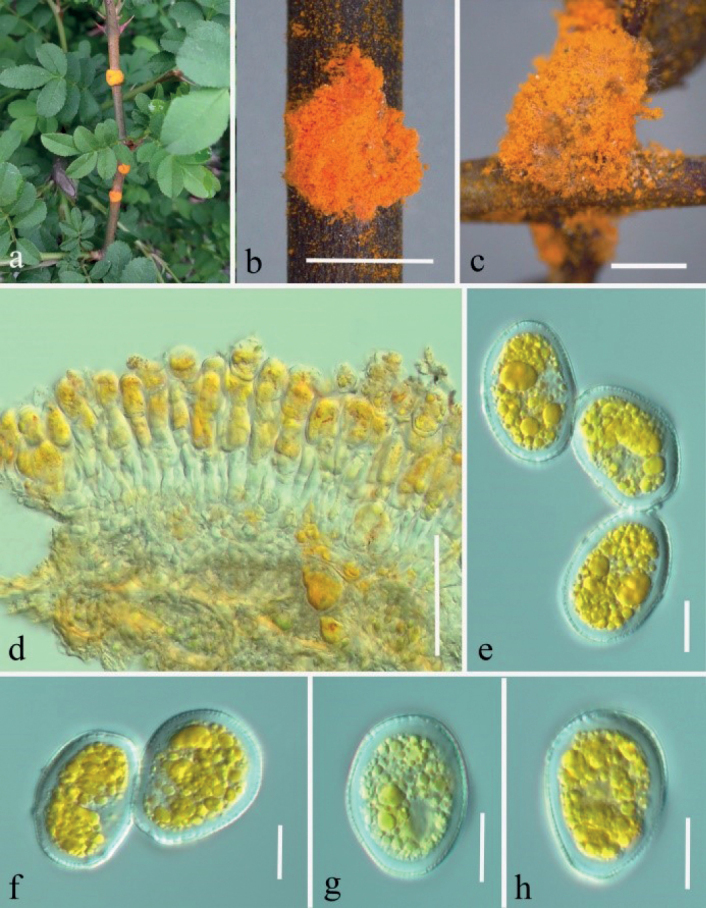
*Phragmidiumrosae-roxburghii* sp. nov. (HGUP21025, holotype) on *Rosaroxburghii***a–c** aecia on stem and leaf pieces. **d** longitudinal section of aecium **e–h** aeciospores. Scale bars: 2 mm (**b–c**); 50 µm (**d**); 10 µm (**e–h**).

**Figure 3.**
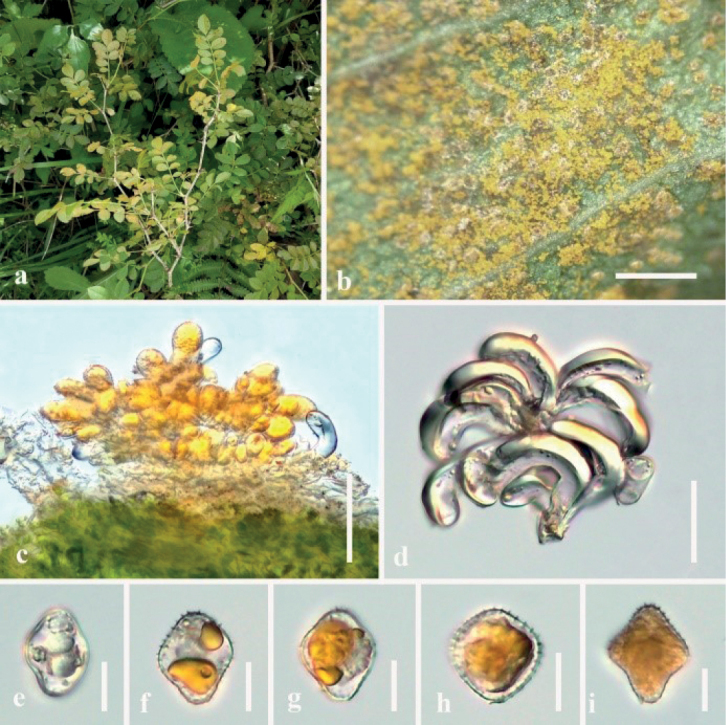
*Phragmidiumrosae-roxburghii* sp. nov. (HGUP21026) on *Rosaroxburghii***a** appearance of infected plants **b** uredinia on a leaf **c** longitudinal section of uredinium **d** paraphyses **e–i** urediniospores. Scale bars: 5 mm (**b**); 50 µm (**c**); 25 µm (**d**); 12.5 µm (**e–i**).

Rust diseases symptoms: In the early stage (March) of rust disease yellowish-orange powdery aecia formed on the stems and petioles on *Rosaroxburghii* and *Rosa* sp., the aecia were scattered, flat oval or nearly round and bordered (Fig. 2). In middle of June (Fig. 3), the upper surface of the lower leaves was turning yellow and orange spots gradually appeared on the under surface caused by uredinia, which are powdery, aggregated but without obvious boundaries.

##### Habitat.

*Rosaroxburghii*, *Rosa* sp.

##### Known distribution.

China, Guizhou Province.

##### Additional material examined.

China. Guizhou Province: Duyun city, 26°45'88"N, 106°98'42"W, 820 m, 22 Jun 2021, on *Rosaroxburghii*, coll. J.E. Sun, HGUP21026; Tongren city, 28°14'09"N, 108°34'03"W, 810 m, 04 Sep 2021, on *Rosaroxburghii*, coll. J.E. Sun, HGUP21027; Guiyang city, 26°44'74"N, 106°58'67"W, 960 m, 27 Mar, 2021, on *Rosa* sp., coll. J.E. Sun, HGUP21028.

##### Notes.

*Phragmidiumrosae-roxburghii* was the first species of *Phragmidium* described on *Rosaroxburghii*. It is easily to distinguish species by its unique square to diamond-shaped urediniospores, since in other *Phragmidium* species the urediniosporas are oval to nearly spherical (Yun et al. 2011; Ono 2012; Zhuang et al. 2012; Yang et al. 2015; Liu et al. 2018, 2019, 2020; Ono and Wahyuno 2019). In phylogeny, this species only kept a close relationship to *Ph.warburgiana* (Fig. 1) but its urediniospores are yellowish to orange-colored different to *Ph.warburgiana* with colorless urediniospores (Ono 2012). We proposed *Ph.rosae-roxburghii* as a new taxon.

#### ﻿ Phragmidium rubi-coreani

Taxon classificationFungiPuccinialesPhragmidiaceae

J.E. Sun & Yong Wang bis sp. nov.

12B1AEFB-6E3E-5149-989B-A3F6B2FDB481

845042

Fig. 4

##### Diagnosis.

*Phragmidiumrubi-coreani* differs to *Ph.barclayi* by teliospores with fewer cells and shorter pedicels.

##### Holotype.

China. Guizhou Province: Guiyang city, 26°45'86"N, 106°98'77"W, 970 m, 11 Apr, 2021, on *Rubuscoreanus*, coll. J.E. Sun, HGUP21029, ITS: OL684822, LSU: OL684833.

##### Etymology.

Referring to the host, *Rubuscoreanus*, on which this species grows.

##### Description.

***Spermogonia***: unknown. ***Aecia*** golden, produced on the abaxial leaf surface, hypophyllous, and 2.5–3.5 mm diam, subglobose to globose, powdery, 2.5–3.5 mm diam. Aeciospores produced in basipetal succession, subglobose, 14–24 × 10–23 µm (mean 19 × 16 μm, n = 30), bright yellow contents, thick-walled, 1.0–4.0 µm, colorless, echinulate; paraphyses clavate, not or weakly incurved, 38–61 μm long, thick-walled, wall 2.0–2.5 μm thick. ***Telia*** hypophyllous, scattered, 0.3–0.5 mm diam, chocolate-brown. Teliospores ellipsoid to cylindrical, 3–5 celled, constricted at the septa, bright orange, chocolate-brown to gray-brown, 29–74 × 14–37 µm (mean 50 × 25 μm, n = 30), thick-walled, wall 1.8–3.5 μm thick, colorless to chocolate-brown; pedicels not swollen at the base, 8–34 μm long, colorless. ***Uredinia*** formed on circular lesions on both sides of the leaves, powdery, yellow distinct, hypophyllous scattered, nearly oval, surrounded by host epidermis, 0.5–1.0 mm diam. Urediniospores: uredo-type, subglobose to oval, produced in basipetal succession, golden, or bright-yellow, 19–27 × 15–25 µm (mean 23 × 20 μm, n = 30), thick-walled, wall 0.8–1.5 µm thick, colorless, densely and minutely echinulate.

**Figure 4.**
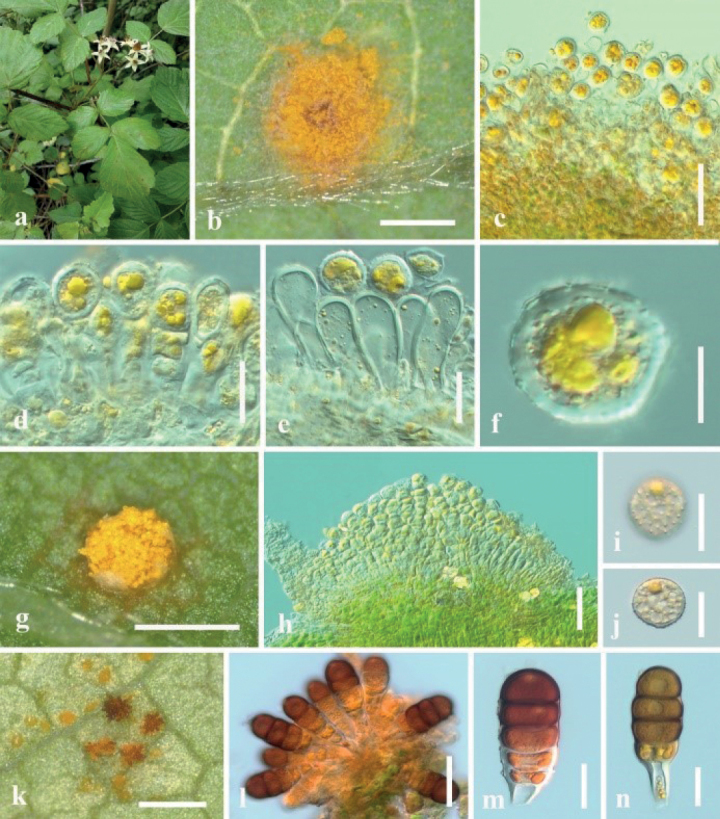
*Phragmidiumrubi-coreani* sp. nov. (HGUP21029, holotype) on *Rubuscoreanus***a** gross features of infected leaves **b** uredinia on a leaf **c–d** longitudinal section of uredinium **e** paraphyses **f** urediniospores **g** aecia on a leaf **h** longitudinal section of aecium **i–j** aeciospores **k** telia on a leaf **l** longitudinal section of telium **m–n** Teliospores. Scale bars: 2 mm (**b**); 1 mm (**g, k**); 50 µm (**c–e, h, l**); 10 µm (**f**); 25 µm (**i–j, m–n**).

Rust diseases symptoms: The golden and powdery aecia were first produced on the underside of leaves. Then, scattered uredinia were formed, orange-colored and forming small round spots on the leaves. Chocolate-brown telia were produced on the leaf remnants (Fig. 4).

##### Habitat.

*Rubuscoreanus*.

##### Known distribution.

China, Guizhou Province.

##### Additional material examined.

China. Guizhou Province: Guiyang city, 27°10'30"N, 106°99'91"W, 830 m, 09 Apr 2021, on *Rubuscoreanus*, coll. J.E. Sun, HGUP21030.

##### Notes.

In the phylogenetic tree, *Phragmidiumrubi-coreani*, *Ph.barclayi* and *Ph.cibanum* formed a branch (Fig. 1). However in morphology, teliospores of *Phragmidiumrubi-coreani* have fewer septa and shorter pedicels (3–5-celled, 8–34 μm long) than *Ph.barclayi* (5–8-celled, 60–150 μm long) and *Ph.cibanum* (5–7-celled, 70–108 μm long) (Liu et al. 2018). Meanwhile, most reported *Phragmidium* taxa produce longer teliospores, such as *Ph.zangdongii* (29–74 × 14–37 µm vs. 82–110 × 23–31 μm); *Ph.kanas* (29–74 × 14–37 µm vs. 134–198 × 19–31 µm); *Ph.potentillae-canadensis* (29–74 × 14–37 µm vs. 48.1–86.8 × 30.1–33.3 µm) than the present species (Yun et al. 2011; Liu et al. 2018; Zhao et al. 2021). Thus, our fungus represented a novel taxon.

#### ﻿ Phragmidium potentillae-freynianae

Taxon classificationFungiPuccinialesPhragmidiaceae

J.E. Sun & Yong Wang bis sp. nov.

9854E361-3682-52BE-95C7-B3926A70F364

845043

Fig. 5

##### Diagnosis.

Different from the related taxa by its urediniospores catenulate, such as *Ph.chayuensis*, *Ph.cibanum* and *Ph.tormentillae*.

##### Holotype.

China. Guizhou Province;, Guiyang city, 26°44'70"N, 106°59'65"W, 801 m, 27 Mar 2021, on *Potentillafreyniana*, coll. J.E. Sun, HGUP21033, ITS: OL684826, LSU: OL684837.

##### Etymology.

Referring to the host, *Potentillafreyniana*, on which the fungus was first found.

##### Description.

***Spermogonia***, ***aecia*** and ***telia*** not observed. ***Uredinia*** produced on the abaxial leaf surface, covering the entire lower surface of the leaves, hypophyllous, nearly oval, powdery, not surrounded by host epidermis, 0.1–1.0 mm diam, on densely orange spot, 0.1–1.0 mm diam. Urediniospores: uredo-type, subglobose to oval, produced in basipetal succession, 19–24 × 18–24 µm (mean 21.5 × 21 μm, n = 30), golden, or bright-yellow; thin-walled, wall 0.4–1.4 µm thick, colorless, densely and minutely echinulate.

**Figure 5.**
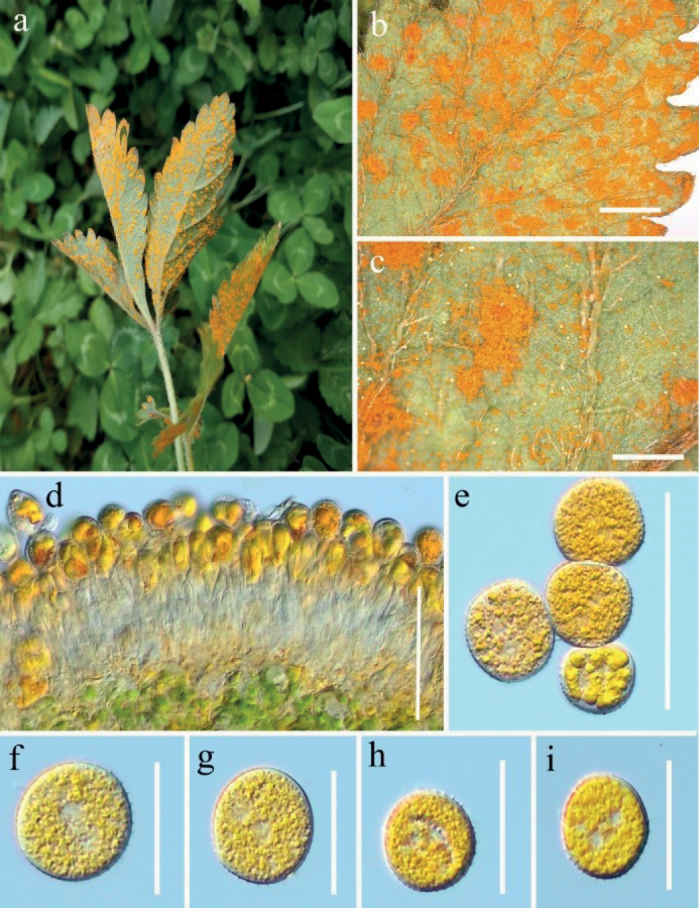
*Phragmidiumpotentillae-freynianae* sp. nov. (HGUP21033, holotype) on *Potentillafreyniana*. **a–c** uredinia on leaves **d** longitudinal section of uredinium **e–i** urediniospores. Scale bars: 2 mm (**b–c**); 50 µm (**d–e**); 25 µm (**f–i**).

Rust diseases symptoms: Large areas of orange powdery uredinia, covering almost the entire lower surface of the leaves, which are aggregated but without obvious boundaries (Fig. 5).

##### Habitat.

*Potentillafreyniana*.

##### Known distribution.

China, Guizhou Province.

##### Notes.

In the phylogenetic tree, *Phragmidiumpotentillae-freynianae* formed a well-supported clade allied to *Ph.duchesneae-indicae* (Fig. 1). Morphologically, its urediniospores are bigger than *Ph.duchesneae-indicae* (21.5 × 21 μm vs. 13–19 × 11–17 µm) (Zhao et al. 2021). The comparison of DNA base composition supports the morphological separation of this fungus as a new species.

#### ﻿ Phragmidium rosae-laevigatae

Taxon classificationFungiPuccinialesPhragmidiaceae

J.E. Sun & Yong Wang bis sp. nov.

C2788B76-560E-5782-AD55-6E005D13C140

845044

Fig. 6

##### Diagnosis.

Different from *Ph.Jiangxiense* mainly because of bigger urediniospores.

##### Holotype.

China. Guizhou Province: Panzhou city, 25°64'56"N, 104°84'35"W, 1800 m, 19 Jul 2021, on *Rosalaevigata*, coll. J.E. Sun, HGUP21036, ITS: OL684829, LSU: OL684840.

##### Etymology.

Referring to the host, *Rosalaevigata*, on which the fungus was first found.

##### Description.

***Spermogonia*** and ***aecia*** not observed. ***Uredinia*** produced on the abaxial leaf surface, hypophyllous, subglobose to globose, powdery, 0.1–0.5 mm diam, yellow, peripherally parphyses, hyaline, 20–31 × 10–17 µm. Urediniospores square to diamond-shaped, oval to nearly spherical, 23–35 × 16–30 µm (mean 29 × 23 µm, n = 30), orange-colored, thick-walled 0.5–2.0 µm thick, colorless, regularly echinulate with stout spines on the surface. ***Telia*** scattered compact, hypophyllous, golden, 0.1–0.5 mm diam. Teliospores (immature) oval, 24–60 × 8–20 µm (mean 50.5 × 25.5 μm, n = 30), with apical papillae (4.0–7.0 μm high, n = 10), too immature to know how many cells, orange-yellow; pedicels swollen at the base, 15–26 μm long, colorless, disconnected easily; wall 0.5–2.0 μm thick.

**Figure 6.**
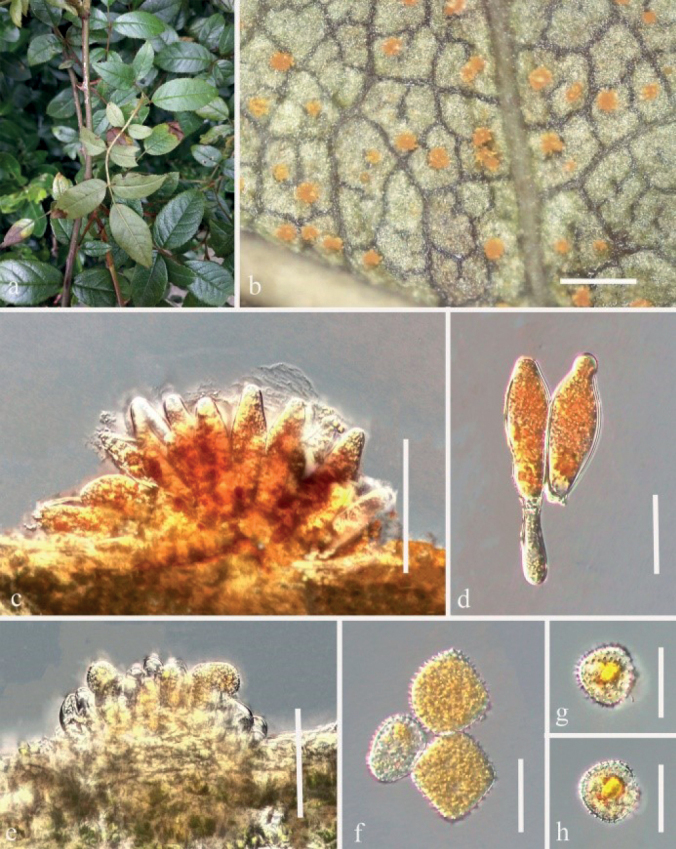
*Phragmidiumrosae-laevigatae* sp. nov. (HGUP21036, holotype) on *Rosalaevigata***a** gross features of infected leaves **b** uredinia and telia on a leaf **c** longitudinal section of telium **d** immature teliospores **e** longitudinal section of uredinium **f–h** urediniospores. Scale bars: 1 mm (**b**); 50 µm (**c, e**); 12.5 µm (**d, f**–**h**).

Rust diseases symptoms: As shown in Fig. 6, Uredinia and telia, which are bright-yellow and powdery are produced almost simultaneously on the lower surface of the yellowing and wilting leaves.

##### Habitat.

*Rosalaevigata*.

##### Known distribution.

China, Guizhou Province.

##### Additional material examined.

China. Guizhou Province: Panzhou city, 25°61'81"N, 104°83'61"W, 1790 m, 19 Jul 2021, on *Rosalaevigata*, coll. J.E. Sun, HGUP21037.

##### Notes.

Phylogenetically, *Phragmidiumrosae-laevigatae* kept a close relationship to *Ph.leucoaecium*, *Ph.japonicum* and *Ph.jiangxiense* (Fig. 1). Morphologically, *Phragmidiumrosae-laevigatae* has bigger urediniospores than *Ph.jiangxiense* (23–35 × 16–30 µm vs. 15–23 × 11–18 μm), but the uredinia and urediniospores of *Ph.leucoaecium* and *Ph.japonicum* were not observed (Liu et al. 2020). The comparison of DNA base composition also supported morphological conclusion. Thus, this fungus was also introduced as one novel taxon herein.

#### ﻿ Phragmidium duchesneae-indicae

Taxon classificationFungiPuccinialesPhragmidiaceae

P. Zhao & L. Cai, Fungal Diversity 5:1–58, 2021

77D32672-61F9-5904-978F-A87AC9C51457

557609

Fig. 7

##### Description.

***Spermogonia***, ***aecia*** and ***telia*** not observed. ***Uredinia*** produced on the abaxial leaf surface, hypophyllous, nearly oval, golden, densely bright orange-yellow, powdery, not surrounding by host epidermis, 0.3–1.2 mm diam, without paraphyses. Urediniospores produced in basipetal succession, mostly globose, 17–22 × 15–20 µm (mean 19.5 × 17.5 μm, n = 30), inclusions yellowish, or bright-yellow; thick-walled, wall 0.7–1.8 µm thick, colorless, densely and minutely echinulate. Telia and teliospores see Zhao et al (2021).

**Figure 7.**
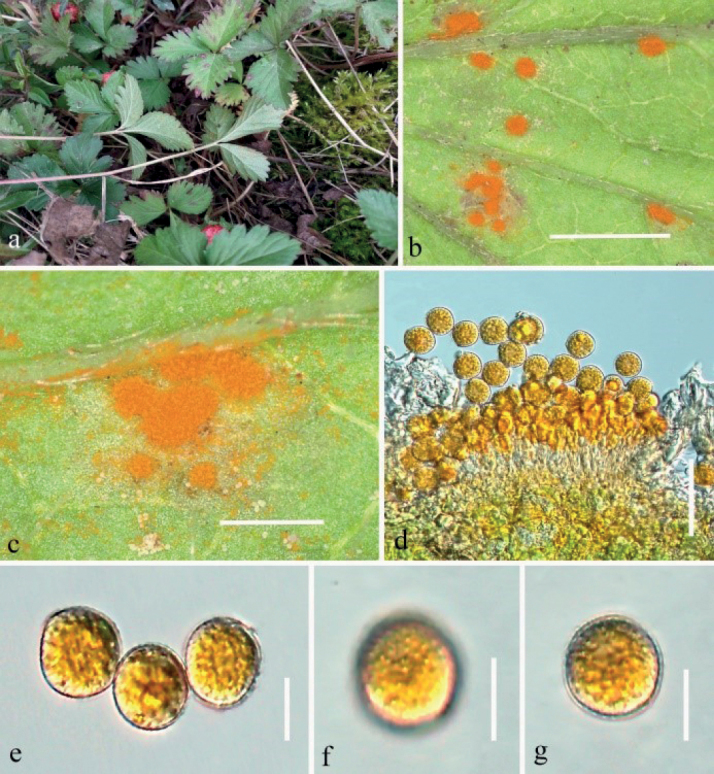
*Phragmidiumduchesneae-indicae* (HGUP21031) on *Duchesneaindica***a–c** uredinia on leaves **d** longitudinal section of uredinium **e–g** urediniospores. Scale bars: 2 mm (**b**); 1 mm (**c**); 50 µm (**d**); 12.5 µm (**e–g**).

##### Habitat.

*Duchesneaindica*

##### Known distribution.

China, Guizhou Province.

##### Material examined.

China. Guizhou Province: Guiyang city, 27°10'30"N, 106°99'91"W, 820 m, 09 Apr 2021, on *Duchesneaindica*, coll. J.E. Sun, HGUP21031; Guiyang city, 27°09'26"N, 106°98'90"W, 734 m, 04 Sep 2021, on *Duchesneaindica*, coll. J.E. Sun, HGUP21032.

##### Notes.

*Phragmidiumduchesneae-indicae* was first reported on *D.indica* by Zhao et al (2021). Our specimen had similar morphology to that described by Zhao et al (2021). GenBank accession numbers (ITS and *LSU*) of *Ph.duchesneae-indicae* have not been released, and our identification is based only on a morphological comparison.

#### ﻿ Phragmidium potentillae

Taxon classificationFungiPuccinialesPhragmidiaceae

(Pers.) P. Karst., Bidrag till Kännedom av Finlands Naturoch Folk, 31: 49, 1879

E5AAFEF4-857A-530F-A5E9-169F68BABC05

206190

Fig. 8

##### Description.

***Spermogonia*** and ***aecia*** not observed. ***Uredinia*** produced on the abaxial leaf surface, hypophyllous, nearly oval, powdery, densely bright orange, nearly oval, surrounding by host epidermis, 0.8–1.5 × 0.4–0.7 mm, and densely bright orange. Urediniospores angular to squarish, oval to nearly globose, produced in basipetal succession, 17–26 × 14–22 µm (mean 21.5 × 18 μm, n = 30), or bright–yellow to orange, immature urediniospores are colorless; thick-walled, wall 0.6–1.3 µm thick, colorless, densely and minutely echinulate. Telia and teliospores see Liu et al (2018).

**Figure 8.**
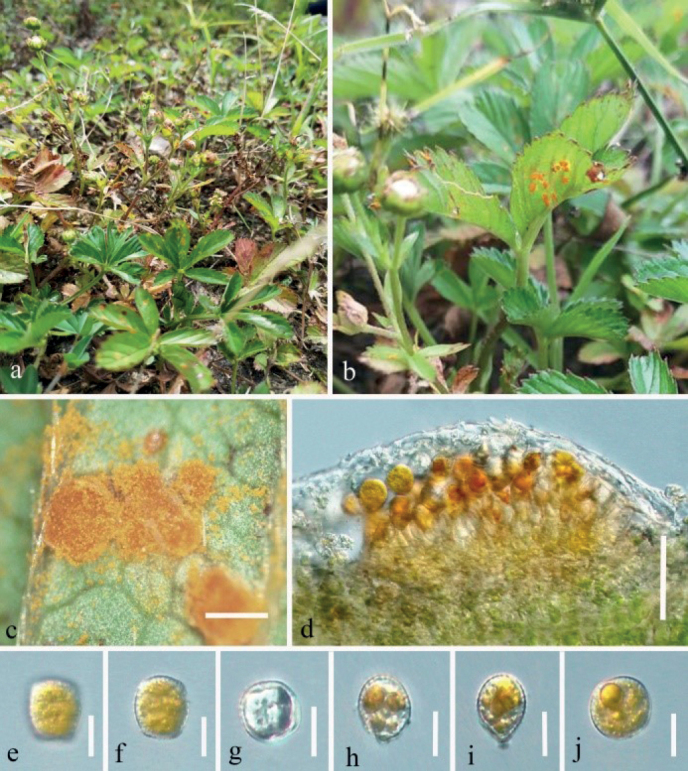
*Phragmidiumpotentillae* (HGUP21034) on *Potentillakleiniana***a–c** uredinia on leaves **d** longitudinal section of uredinium **e–j** urediniospores. Scale bars: 1 mm (**c**); 50 µm (**d**); 12.5 µm (**e–j**).

##### Habitat.

*Potentillakleiniana*

##### Known distribution.

China: Guizhou Province, Qinghai Province, Sinkiang Province; USA, the United Kingdom, Australia, Tasmania and Japan.

##### Material examined.

China. Guizhou Province: Guiyang city, 27°09'26"N, 106°98'90"W, 730 m, 22 Jun 2021, on *Potentillakleiniana*, coll. J.E. Sun, HGUP21034.

##### Notes.

In the phylogenetic tree, HGUP21034 clustered with two sequences of specimens of *Phragmidiumpotentillae* (Fig. 1). The uredinia of *P.potentillae* described by Liu et al (2018), as 0.2–0.8 mm diam, smaller than in the specimen examined, 0.8–1.5 × 0.4–0.7 mm, the urediniospores mostly globose and echinulate, (18–25 × 15–21 μm vs. 17–26 × 14–22 µm).

#### ﻿ Phragmidium barnardii

Taxon classificationFungiPuccinialesPhragmidiaceae

Plowr. & G. Winter, Revue Mycologique Toulouse 8 (32): 208 (1886)

ABF95ED4-5864-5C3B-AB24-CFFAE32D5130

249450

Fig. 9

##### Description.

***Spermogonia***, ***aecia*** and ***telia*** not observed. Uredinia produced on the abaxial leaf surface, hypophyllous, scattered to gregarious, oval to globose, orange, powdery, 0.1–1.0 mm diam, with hyaline and curved paraphyses, 26–39 × 10–13 µm. Urediniospores orange, 16–19 × 15–18 µm (mean: 17.5 × 16.5 µm, n = 30), nearly globose; thick-walled 1.3–2.2 µm, colorless, regularly echinulate with stout spines.

**Figure 9.**
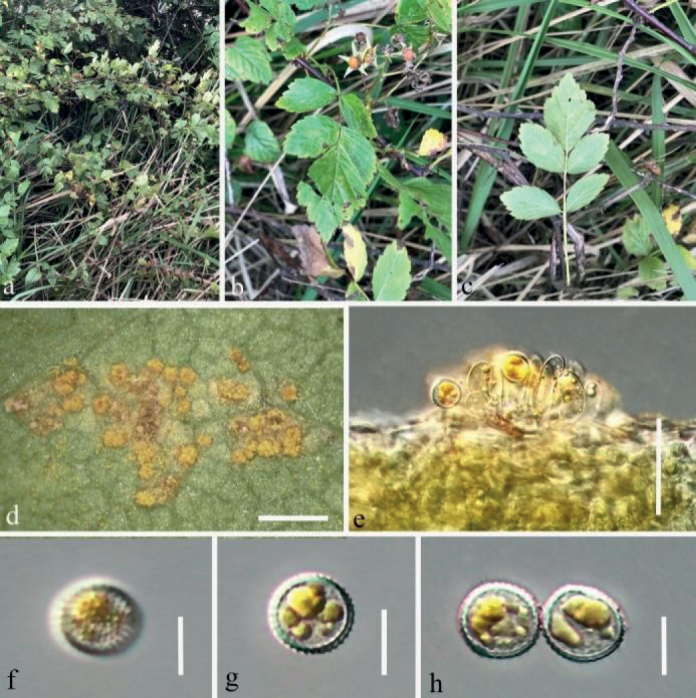
*Phragmidiumbarnardii* (HGUP21035) on *Rubus* sp. **a–d** uredinia on leaves **e** longitudinal section of uredinium **f–h** urediniospores. Scale bars: 1 mm (**d**); 50 µm (**e**); 12.5 µm (**f–h**).

##### Habitat.

*Rubus* sp.

##### Known distribution.

China, Guizhou Province; South Africa.

##### Material examined.

China. Guizhou Province: Duyun city, 27°26'05"N, 107°38'91"W, 870 m, 26 Jun 2021, on *Rubus* sp., coll. J.E. Sun, HGUP21035.

##### Notes:

*Phragmidiumbarnardii* was first reported on *Rubus* sp. by Winter (1886). Its DNA data was established by McTaggart et al (2016), although without description of morphological characteristics. We confirmed the specimens (HGUP21035) as *Ph.barnardii*, through phylogenetic analyse with DNA data from McTaggart et al. (2016).

## ﻿Discussion

More than 70 *Phragmidium* species have been described in China, while many species without molecular data (Cummins 1931; Arthur 1934; Wahyuno et al. 2001; Cummins and Hiratsuka 2003; Zhuang et al. 2012; Yang et al. 2015; Ali et al. 2017). Recently, morphology and molecular data were gradually combined and used to describe the diversity of species in *Phragmidium* (Liu et al. 2018, 2019, 2020; Zhao et al. 2021). In the study, the four novel and three known species of *Phragmidium* were delineated based on phylogeny of the ITS and *LSU* gene regions and on morphological features.

The host plants of *Ph.punjabense*, *Ph.warburgianum*, *Ph.rosae-rugosae*, *Ph.rosae-laevigatae* and *Ph.rosae-roxburghii* all belong to *Rosa*, but *Ph.potentillae-freynianae* and *Ph.potentilla* occur on *Potentilla* sp. while *Ph.rubi-coreani* and *Ph.barnardii* occur on *Rubus* sp. However, the hosts of species with close phylogenetic relationships were not necessarily in the same genus. *Phragmidiumpotentilla* can be found on three plants (*P.chinensia*, *P.kleiniana* and *P.virgata*), and *Ph.rosae-roxburghii* can be parasitic on two *Rosa* plants (*Rosaroxburghii* and *Rosa* sp.). It might mean that host jumps also shaped the diversity of *Phragmidium*, like Pucciniales (McTaggart et al. 2016).

*Phragmidiumleucoaecium* (BJFCR02118 and BJFCR02116), *Ph.japonicum* (HMAS41585), *Ph.jiangxiense* (BJFCR03452 and BJFCR03453) and *Ph.rosae-laevigatae* (HGUP21036 and HGUP21037) from *Rosa* formed a phylogenetic lineage, while three of the latter from the same host (*Rosalaevigata*) (Liu et al. 2020). This may be explained by geographical distribution, geography, climate, etc., but contradicts the concept of obligatory parasitism. We could guess that their hosts might not reflect taxonomic status of *Phragmidium*. Interestingly, *Phragmidiumtibeticum*, *Ph.sikangense* and *Ph.shensianum* were named according to the collection locations (Dai 1979; Chen 2009). Their nomenclatures contradict the concept of obligatory parasitism for rust fungi, although might be easy to be understanding.

## Supplementary Material

XML Treatment for Phragmidium rosae-roxburghii

XML Treatment for Phragmidium rubi-coreani

XML Treatment for Phragmidium potentillae-freynianae

XML Treatment for Phragmidium rosae-laevigatae

XML Treatment for Phragmidium duchesneae-indicae

XML Treatment for Phragmidium potentillae

XML Treatment for Phragmidium barnardii
